# Coast to coast: High genomic connectivity in North American scoters

**DOI:** 10.1002/ece3.5297

**Published:** 2019-06-03

**Authors:** Sarah A. Sonsthagen, Robert E. Wilson, Philip Lavretsky, Sandra L. Talbot

**Affiliations:** ^1^ US Geological Survey Alaska Science Center, 4210 University Dr. Anchorage Alaska; ^2^ Department of Biological Sciences University of Texas at El Paso El Paso Texas

**Keywords:** connectivity, dispersal, genetic structure, *Melanitta*, population genomics, sea ducks, waterfowl

## Abstract

Dispersal shapes demographic processes and therefore is fundamental to understanding biological, ecological, and evolutionary processes acting within populations. However, assessing population connectivity in scoters (*Melanitta *sp.) is challenging as these species have large spatial distributions that span remote landscapes, have varying nesting distributions (disjunct vs. continuous), exhibit unknown levels of dispersal, and vary in the timing of the formation of pair bonds (winter vs. fall/spring migration) that may influence the distribution of genetic diversity. Here, we used double‐digest restriction‐associated DNA sequence (ddRAD) and microsatellite genotype data to assess population structure within the three North American species of scoter (black scoter, *M. americana*; white‐winged scoter, *M. deglandi*; surf scoter, *M. perspicillata*), and between their European congeners (common scoter, *M. nigra*; velvet scoter, *M. fusca*). We uncovered no or weak genomic structure (ddRAD *Φ*_ST_ < 0.019; microsatellite *F*_ST_ < 0.004) within North America but high levels of structure among European congeners (ddRAD *Φ*_ST_ > 0.155, microsatellite *F*_ST_ > 0.086). The pattern of limited genomic structure within North America is shared with other sea duck species and is often attributed to male‐biased dispersal. Further, migratory tendencies (east vs. west) of female surf and white‐winged scoters in central Canada are known to vary across years, providing additional opportunities for intracontinental dispersal and a mechanism for the maintenance of genomic connectivity across North America. In contrast, the black scoter had relatively elevated levels of divergence between Alaska and Atlantic sites and a second genetic cluster found in Alaska at ddRAD loci was concordant with its disjunct breeding distribution suggestive of a dispersal barrier (behavioral or physical). Although scoter populations appear to be connected through a dispersal network, a small percentage (<4%) of ddRAD loci had elevated divergence which may be useful in linking areas (nesting, molting, staging, and wintering) throughout the annual cycle.

## 1 INTRODUCTION

Genetic connectivity among populations has a large influence on the persistence of populations via the enrichment of genetic diversity within populations, thereby limiting the deleterious effects of inbreeding and increasing resiliency of populations to environmental stochasticity. Dispersal also shapes demographic processes by forming linkages among populations that can promote stability via immigration and ultimately affect population vital rates (Lowe & Allendorf, 2010). Characterizing dispersal, therefore, is fundamental to understanding biological, ecological, and evolutionary processes acting within populations (Kendrick et al., 2017) and is often a component in species management strategies. Dispersal of individuals across the landscape, the magnitude of exchange, and dispersal distance are not only determined by physical landscape features, but also shaped by behavioral tendencies of individuals and populations (e.g., philopatry and density‐dependent dispersal). Detecting dispersal events directly is challenging, especially at scales relevant to assessments of connectivity (Hedgecock, Barber, & Edmands, 2007; Palumbi, 2003), limiting our ability to make inferences regarding the role dispersal plays on mechanisms regulating population and community dynamics and evolutionary processes. Assessments of connectivity are particularly difficult for species that nest at high latitudes, as they often have large distributions across the annual cycle that span remote (uninhabited) landscapes, further reducing the detectability of dispersal events and ability to evaluate the contribution of dispersers to the population (e.g., gene flow).

**Figure 1:**
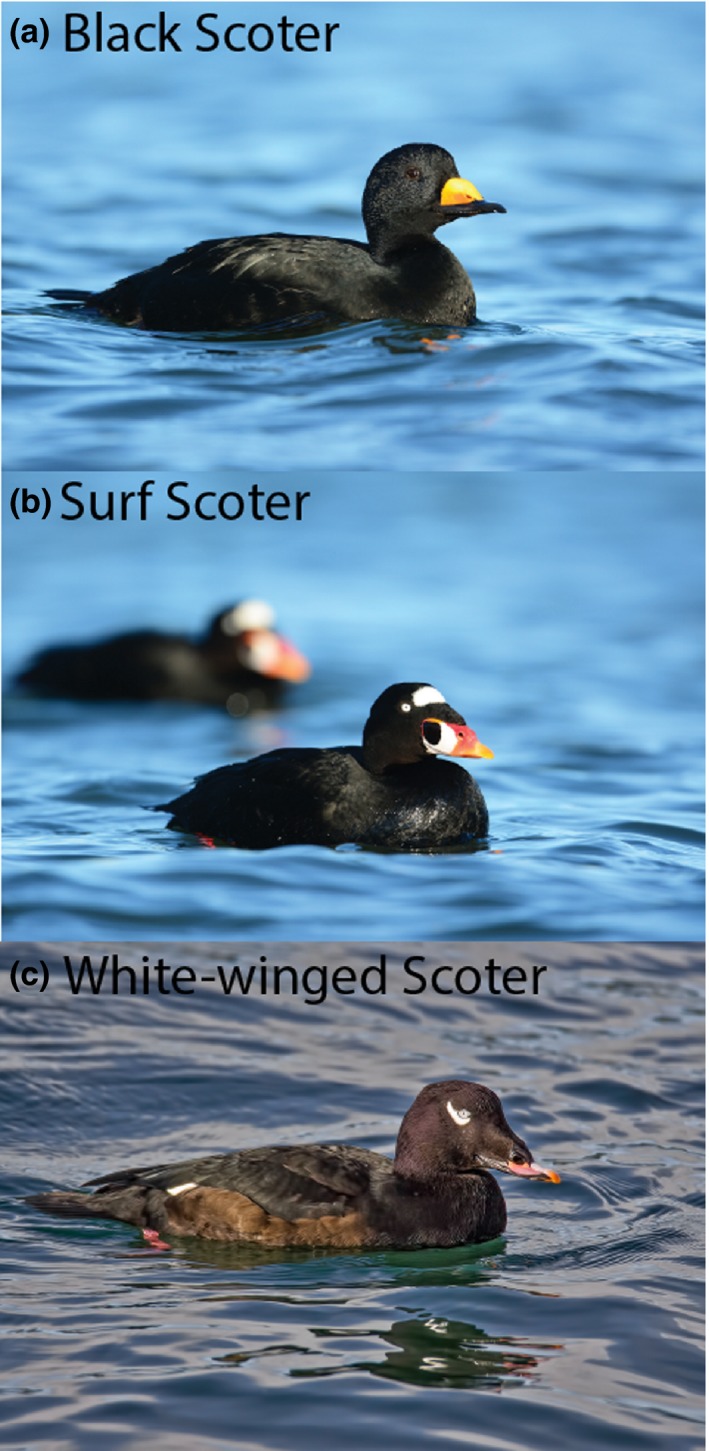
Photographs of the three North American scoters (*Melanitta* sp.): black scoter (a; *M. americana*), surf scoter (b; *M*. *perspicillata*), and white‐winged scoter (c; *M. deglandi*). Photo credits: R. Askren (US Geological Survey) and A. Wilson (Nature's Pics Online, made available under CC BY‐SA 3.0 license)

**Figure 2:**
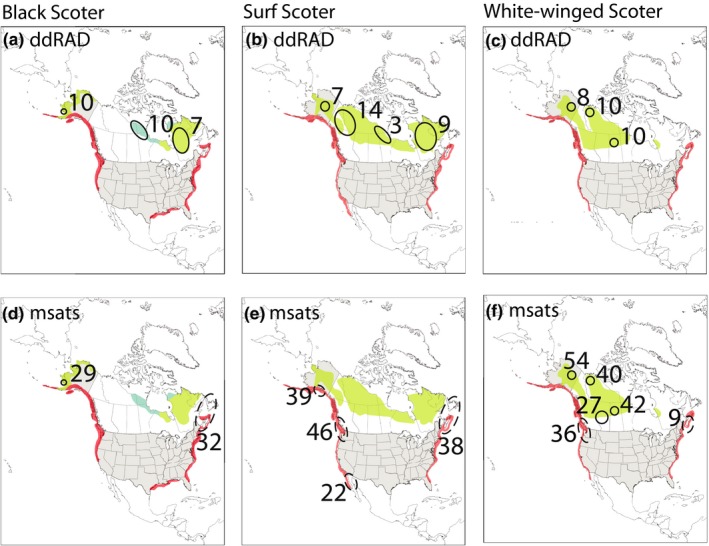
Range (breeding: green and blue; wintering: orange) with location and number of samples assayed of black scoter (a, d), surf scoter (b, e), and white‐winged scoter (c, f) in North America using double‐digest restriction‐associated DNA sequence (ddRAD; a–c) and microsatellite genotype (msats; d–f) methods. Sample locations representing breeding areas are designated with solid circles and nonbreeding locales with dashed circles. Blue represents newly identified breeding areas (see Sea Duck Joint Venture, 2015)

Scoters (*Melanitta *sp.) are a group of sea ducks that inhabit Holarctic waters using freshwater or brackish lakes and ponds for nesting (Anderson et al., 2015; Bordage & Savard, 2011; Brown & Fredrickson, 1997; Takekawa et al., 2011). In North America, there are three species of scoters (black scoter *M. americana*, surf scoter *M. perspicillata*, and white‐winged scoter *M. deglandi*; Figure 1) and three species/populations occurring in Eurasian (common scoter *M. nigra*, velvet scoter *M. fusca*—Europe, and white‐winged scoter *M. deglandi*—Asia). In North America, scoter nesting distribution extends throughout most of the boreal and arctic zones of Alaska and Canada with surf and white‐winged scoters having the most extensive breeding range (Figure 2; Baldassarre, 2014). In Eurasia, scoters in general can be found nesting throughout most of northern Europe eastward through Russia to Siberia. Populations on both continents winter mainly in coastal regions: Atlantic and Pacific oceans along with the Great Lakes in North America; and primarily coastal areas of Black and Caspian seas with Asian white‐winged scoters wintering in the northern Pacific Ocean (BirdLife International, 2018a, 2018b, 2018c).

Scoters exhibit delayed breeding, low reproductive output with annually variable reproductive success, and are long‐lived, which may make them more sensitive to factors that influence adult survival, such as environmental change (e.g., human disturbances or climate change; Anderson et al., 2015, Bordage & Savard, 2011, Brown & Fredrickson, 1997) and overharvest (Allendorf & Hard, 2009; Stott & Olson, 1972). Scoters are in apparent decline across their ranges, which has facilitated a Red List classification as vulnerable for velvet scoter (*M. fusca*) and near‐threatened for black scoter (*M. americana*; BirdLife International, 2016a, 2016b; BirdLife International, 2018a, 2018b, 2018c); status is unknown for common scoter (*M. nigra*). The influence of environmental change, harvest pressure, and other mechanisms regulating population dynamics, however, is poorly known for all scoter species. Scoter population recovery following declines, regardless of cause, may be longer when compared to other waterfowl (e.g., dabbling ducks) due to life‐history characteristics of this group, and therefore, estimating levels of genetic structure among scoter populations is needed (Sea Duck Joint Venture, 2015). Data regarding genetic connectivity will increase our understanding of species biology and provide critical information for predicting how these species may respond to future environmental and other disturbances.

Here, we present the first assessment of genomic connectivity within North American scoters to provide much needed insight into distribution of genetic variation across the landscape, allowing us to infer evolutionary dispersal (i.e., gene flow) and ultimately linkages among populations. North American scoters vary in their nesting distributions (Figure 2) as well as timing of the formation of pair bonds (winter sites vs. during migration), and therefore, patterns of genetic variation may differ across species (Table 1). Specifically, the black scoter exhibits a discontinuous range which may limit gene flow and result in genetic discontinuities. Conversely, the surf scoter has a continuous distribution suggestive of fewer inter‐regional barriers to dispersal. Differences in the timing of mate selection have been hypothesized to account, at least in part, for differential patterns of genetic structure in geese (Ely & Scribner, 1994; Ely, Wilson, & Talbot, 2017; Wilson, Ely, & Talbot, 2018). Within scoters, black and surf scoter (and ducks in general) form pair bonds on the wintering grounds, which typically comprise individuals from multiple nesting locales, thus providing an opportunity for increased gene flow. Conversely, white‐winged scoters pair during spring migration or soon after arrival at the nesting area (Brown & Fredrickson, 1997) and therefore may exhibit genetic structure among migratory pathways. This evidence suggests that scoters possess ecological and behavioral characteristics that have been shown to facilitate population structure as well as promote genetic connectivity across breeding regions in other waterfowl species (Sonsthagen, Talbot, Scribner, & McCracken, 2011; Wilson et al., 2018; Wilson, Gust, Petersen, & Talbot, 2016).

**Table 1:** Selected life‐history attributes of North American scoters and hypothesized potential for population structuring. Species attributes that are unknown are denoted with a question mark

Species	Nest site fidelity	Timing of pairing	Pair‐bond stability	Breeding distribution	Inter‐regional dispersal	Potential for structure
Black scoter	?	Winter	?	Disjunct	Low	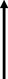
White‐winged scoter	Female	Spring–Summer	Potential reformation	Partially disjunct	High	
Surf scoter	Female?	Winter	Potential reformation	Continuous	High	

For this study, first, we used genome‐wide scans (double‐digest restriction‐site‐associated DNA sequences; ddRAD) and microsatellite genotype data to assess population genomic structure of the three North American scoter species across four regions (Alaska, Pacific, Central, and Atlantic). Second, we assessed levels of intercontinental dispersal between North American and European forms as vagrancy from migratory routes has been observed in all scoters (e.g., black scoter and surf scoter are observed in Europe, Scandinavia, and Russia; Anderson et al., 2015, Bordage & Savard, 2011). Third, as waterfowl are known for high hybridization rates (Gillham & Gillham, 1998; Kraus et al., 2012), we searched for evidence of potential hybridization or introgression among scoters. Lastly, as information regarding annual linkages among areas is needed, we sought to identify high‐resolution (i.e., divergent) loci with a strong potential for nesting area identification (e.g., Ruegg et al., 2014).

## 2 METHODS AND MATERIALS

### 2.1 Taxonomy and sampling

Of the five species of scoter (depending on taxonomic authorities), three occur in North America (black scoter, white‐winged scoter, surf scoter) and two in Eurasia (common scoter, velvet/white‐winged scoter). Traditionally, black scoter and common scoter were treated as conspecifics and were only recently regarded as different species, as they show distinct phenotypic characters (Chesser et al., 2016). Similarly, taxonomic relationships between white‐winged scoter and velvet scoter have varied over time; currently, they are considered conspecific by the American Ornithological Society and separate species by British Ornithological Union.

Samples were opportunistically collected as part of other research efforts, across the ranges of black, white‐winged, and surf scoters in North America (Figure 2), as were representatives of the two European forms, common scoter (ddRAD *N* = 5; msats *N* = 19) and velvet/white‐winged scoter (ddRAD *N* = 4; msats *N* = 20). Samples were grouped into four broad North American regions: Alaska, Pacific, Central, and Atlantic. Regions do not necessarily correspond to migratory flyways, as some sampled locales are comprised of birds that use different flyways (see Discussion). See Sonsthagen, Pierson, Wilson, and Talbot (2019) for additional sample location information.

### 2.2 Library preparation and de‐multiplexing

Genomic DNA was extracted from blood or tissue using a DNeasy Blood and Tissue kit following the manufacturer's protocols (Qiagen). Extractions for the ddRAD protocol were quantified using a NanoDrop 2000 Spectrophotometer (Thermo Fisher Scientific Inc.) to ensure a minimum concentration of 0.02 µg/µl and visualized on an 1% agarose gel for high molecular weight bands. Library preparation for multiplexing followed steps outlined in (DaCosta & Sorenson, 2014; also see Lavretsky et al., 2015, 2016). In brief, we used 10 U of each restriction enzyme SbfI and EcoRI to digest ~1 μg of genomic DNA. For de‐multiplexing reads, we ligated Illumina TruSeq compatible adapters and barcodes to fragmented genomes. Fragments of 300–450 bp (including adapters) were size‐selected using gel electrophoresis (2% low‐melt agarose) and purified using a MinElute Gel Extraction Kit (Qiagen). Size‐selected fragments were then PCR amplified with Phusion High‐Fidelity DNA polymerase (Thermo Scientific), and the amplified products were purified using a 1.8x concentration of AMPure XP beads (Beckman Coulter, Inc.). The concentration of purified PCR products was estimated using quantitative PCR and Illumina library quantification kits (KAPA Biosystems). Finally, the samples were pooled in equimolar concentrations, and 150 base pair, single‐end sequencing was completed on an Illumina HiSeq 2500 at the Tufts University Core Genomics Facility. Raw Illumina reads have been accessioned on National Center for Biotechnology Information (NCBI) Sequence Read Archive (BioProject PRJNA541567, accessions SAMN11587829–115187923); see Sonsthagen et al. (2019) for ddRAD accession information by sample.

Raw Illumina reads were de‐multiplexed and processed using the computational pipeline described by DaCosta and Sorenson (2014; Python scripts available at http://github.com/BU-RAD-seq/ddRAD-seq-Pipeline) and following steps outlined in Lavretsky et al. (2015). The pipeline clusters de‐multiplexed and filtered reads into putative loci based on sequence similarity and genomic position as determined by BLAST, aligns reads within each putative locus, and infers genotyping for individual samples at each locus. Briefly, low‐quality reads were filtered and identical reads collapsed for each sample. Next, sequences were clustered into putative loci using the UCLUST function in USEARCH v. 5 (Edgar, 2010) with an –id setting of 0.85. Chromosomal positions across markers were determined by comparing putative loci sequences to the mallard genome (Kraus et al., 2011; Huang et al., 2013; chromosomal assembly provided by T. Farault) using BLASTN v. 2 (Altschul, Gish, Miller, Myers, & Lipman, 1990). Clusters with identical or nearly identical BLAST hits (i.e., aligned to ± 50 bp on the same reference genome) were combined, which has been shown to minimize error associated with imposing a more arbitrary similarity threshold (see Harvey et al., 2015). Reads within each cluster (i.e., putative locus) were aligned using MUSCLE v. 3 (Edgar, 2004), and samples were genotyped using the Python script *RADGenotypes.py*. Genotypes were scored as homozygous if > 93% of sequence reads were consistent with a single haplotype, whereas heterozygotes were scored if a second haplotype was represented by at least 29% of reads; or if a second haplotype was represented by 20%–29% of reads and the haplotype was present in other individuals. Loci were also “flagged” if the number of single nucleotide polymorphisms (SNPs) were >10, and if >3 SNPs showed strong linkage. This information is produced in the “clustersummary.out” output file of the genotyping step of the DaCosta and Sorenson (2014) pipeline. We used Geneious (Biomatters Inc.) to manually check and edit loci. Doing so, allowed for the retention of many loci with insertions/deletions or high levels of polymorphism. Final datasets consisted of markers that had on average <10% missing genotypes.

Final output files (e.g., FASTA, NEXUS, and ADMIXTURE) were generated with custom python scripts that set a higher minimum sequencing depth to score an allele (Lavretsky et al., 2016). Specifically, to limit any biases due to sequencing error and/or allelic dropout, alleles with <5x coverage were scored as missing, such that a minimum of 10 reads was required to score a locus as heterozygous. Due to female heterogamy at sex chromosomes (Females = ZW, Males = ZZ), and our overarching goal to understand population structure among the species, all analyses were restricted to autosomal markers only. Given that a higher number of loci are expected to be retained for within species than across species datasets, we aimed to test whether an increase in ddRAD markers provided additional resolution. Furthermore, because waterfowl readily hybridize across genera (Ottenburghs, Ydenberg, Hooft, Wieren, & Prins, 2015), combining all five species facilitates our ability to determine whether individuals of hybrid ancestry exist in our dataset. Consequently, a total of four ddRAD datasets were analyzed that included per‐species alignments, as well as an alignment of all five species.

### 2.3 Microsatellite genotyping

Genomic DNA was extracted following Medrano, Aasen, and Sharrow (1990), with modifications described in Sonsthagen, Talbot, and White (2004). Extractions were quantified using fluorometry and diluted to 50 ng/ml working solutions. Genotype data were collected from 11 microsatellite loci: ANAS2323 (K.T. Scribner unpublished: F:ATTGGAGATTTTCAGGACG; R:AGGGAACTGATGCCCCA); Aph02, Aph4, Aph7, Aph8 (Maak, Wimmers, Weigend, & Neumann, 2003); Bca11, Hhi3 (Buchholz, Pearce, Pierson, & Scribner, 1998); CRG (A. Baker, unpublished; see Wilson et al., 2016); Sfi10, Sfi11 (Libants et al. unpublished; GenBank accession nos AF180500 and AF180501, respectively); and Smo7 (Paulus & Tiedemann, 2003). Polymerase chain reaction (PCR) amplifications and thermocycler conditions followed Talbot et al. (2011). In addition, 10% of the samples were extracted, amplified, and genotyped in duplicate for quality control. No inconsistencies in genotype scores were observed between replicates. See Sonsthagen et al. (2019) for microsatellite genotype data.

### 2.4 Population structure and diversity

Within and among species, we calculated composite pairwise estimates of relative divergence (*Φ*_ST_) across ddRAD autosomal markers in the R package PopGenome (Pfeifer, Wittelsbürger, Ramos‐Onsins, & Lercher, 2014) using concatenated datasets, and with insertion/deletion positions treated as missing. Given our goal of testing genetic structure across North America, within‐species analyses included comparisons of samples grouped into four regions: Alaska, Pacific, Central, and Atlantic (Figure 2). Finally, nucleotide diversity (*π*) was estimated in the R package PopGenome (Pfeifer et al., 2014) for chromosomally concatenated ddRAD autosomal loci.

We calculated allelic richness, observed, and expected heterozygosities, Hardy–Weinberg equilibrium (HWE), and linkage disequilibrium (LD) for microsatellite loci in FSTAT 2.9.3 (Goudet, 1995). Pairwise estimates of genetic structure (*F*_ST_ and* R*_ST_) within and among species were calculated in Arlequin 3.1 (Excoffier, Laval, & Schneider, 2005). Tests for HWE, LD, and *F*_ST_ based on microsatellite data were corrected for multiple comparisons using Bonferroni correction (*α* = 0.05).

For ddRAD data, maximum‐likelihood estimates of population assignments for each individual were obtained with ADMIXTURE v.1.3 (Alexander & Lange, 2011; Alexander, Novembre, & Lange, 2009). ADMIXTURE analyses were conducted twice, either with (a) all biallelic single nucleotide polymorphisms (SNPs), excluding singletons (i.e., rare SNPs observed in only one individual), or (b) a single randomly selected biallelic SNP per locus. For both analyses, singletons were excluded and no a priori assignment of individuals to populations or species was included. SNPs were formatted for analyses using plink v. 1.07 (Purcell et al., 2007) and following steps outlined in Alexander, Novembre, and Lange (2012). Separate analyses were done for each scoter species and with all scoter species included in a single analysis. Each ADMIXTURE analysis was run with a 10‐fold cross‐validation and with a quasi‐Newton algorithm employed to accelerate convergence (Zhou, Alexander, & Lange, 2011). To limit possible stochastic effects from single analyses, we ran 100 iterations per analysis and at each population of *K* (from *K* of 1–10). Each analysis used a block relaxation algorithm for point estimation and terminated once the change (i.e., delta) in the log‐likelihood of the point estimations increased by <0.0001. The optimum *K* was based on the average of cross‐validation (CV) errors across the 100 analyses per *K*; however, additional *K*s were analyzed for further population structure resolution (Janes et al., 2017). The program CLUMPP v.1.1 (Jakobsson & Rosenberg, 2007) was then used to determine the robustness of the assignments of individuals to populations at each *K*. First, the R program PopHelper (Francis, 2017) was used to convert ADMIXTURE outputs into CLUMPP input files at each *K*. In CLUMPP, we employed the large greedy algorithm and 1,000 random permutations. Final ADMIXTURE proportions for each *K* and per sample assignment probabilities (*Q* estimates; the log‐likelihood of group assignment) were based on CLUMPP analyses of all 100 replicates per *K*.

For the microsatellite data, we used the Bayesian clustering program, STRUCTURE 2.3.2 (Hubisz, Falush, Stephens, & Pritchard, 2009; Pritchard, Stephens, & Donnelly, 2000), to assign individuals to clusters based on their microsatellite allelic frequencies and infer the occurrence of genetic structure without a priori knowledge of putative populations. Separate analyses were done for each scoter species and with all scoter species included in a single analysis. Data were analyzed using an admixture model assuming correlated frequencies and sample location information as a prior with a 100,000 burn‐in period, 1,000,000 Markov chain Monte Carlo iterations, and number of possible populations (*K*) ranging from 1 to 8; the analyses were repeated 10 times to ensure consistency across runs. We followed the method of Evanno, Regnaut, and Goudet (2005) to determine the most likely number of clusters given the data.

## 3 RESULTS

### 3.1 ddRAD dataset

For within‐species analyses based on traditional species taxonomies (i.e., both North American and Eurasian forms of black scoter and white‐winged scoter, and surf scoter), we recovered 3,487–3,999 ddRAD autosomal markers, comprising ~500K bp and ~15,000 SNPs (Table 2). Final datasets comprised loci with an average median sequencing depth of 78–124 reads per locus per individual, and 92%–97% of alleles scored per individual per locus (Table 2). Among traditional species, a total of 2,224 ddRAD autosomal markers passed filter (304,776 bp; 20,041 SNPs), with an average median sequencing depth of 138 reads per locus per individual, and an average of 96% of alleles scored per individual per locus (Table 2).

**Table 2:** The total number of isolated and pass‐filtered ddRAD autosomal markers, base‐pairs, and biallelic SNPs within‐ and between‐species datasets

	No. of ddRAD Markers	Base‐pairs	Num. of SNPs	Num. of biallelic SNPs	Avg. median sequencing depth (Range)	% of scored alleles (Range)
Black scoter (*N* = 32)	3,692	493,434	15,655	10,624	104 (23–269)	92 (76–99)
Surf scoter (*N* = 33)	3,999	509,937	15,310	11,194	124 (25–1,184)	97 (88–99)
White‐winged scoter (*N* = 32)	3,487	457,961	13,894	9,857	78 (25–401)	96 (89–99)
All scoters (*N* = 97)	2,224	304,776	20,041	13,065	138 (30–1,716)	96 (81–99)

Note

Average median and range of sequencing depth (i.e., number of reads per locus per individual) and percent (%) of alleles scored per dataset, with ranges, are given. Sample sizes (*N*) are in parentheses.

### 3.2 Population structure and diversity estimates

**Figure 3:**
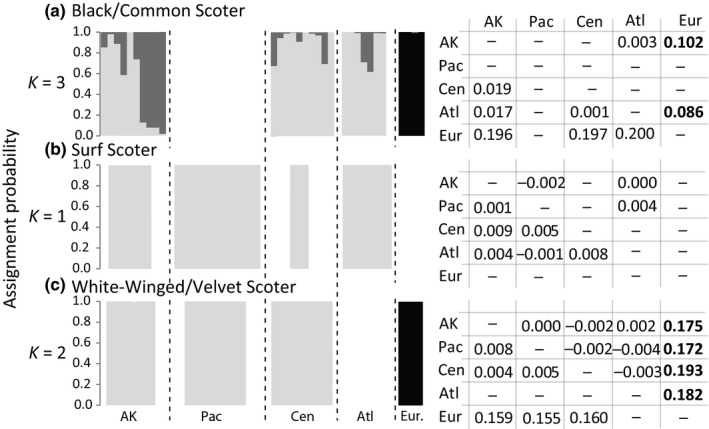
Average assignment probabilities of black/common scoter (a), surf scoter (b), and white‐winged/velvet scoter (c) individuals from sampled regions (AK—Alaska; Pac—Pacific; Cen—Central; Atl—Atlantic; Eur—Europe) into two or three clusters inferred from ddRAD data in ADMIXTURE using all biallelic single nucleotide polymorphic sites along with pairwise estimates of genetic structure (ddRAD *Φ*_ST_ below the diagonal; microsatellite *F*_ST_ above the diagonal) among regions. Significant comparisons after Bonferroni correction (microsatellite data) are in bold text. Pairwise comparisons for white‐winged scoter based on microsatellite data are not presented for nonbreeding and Alberta locales in the Pacific region

**Figure 4:**
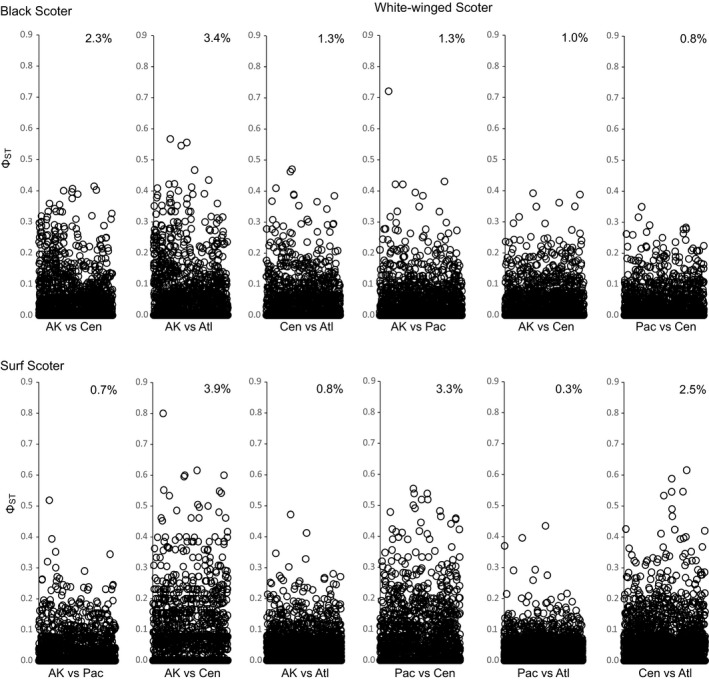
Plots of pairwise *Φ*_ST_ values by double‐digest restriction‐associated sequence (ddRAD) assayed from black scoters, surf scoters, and white‐winged scoters sampled from four regions in North America: Alaska (AK), Pacific (Pac), Central (Cen), and Atlantic (Atl). The percent of loci with an elevated *Φ*_ST_ (> 0.2) is listed for each comparison: 3,692 ddRAD loci were analyzed for black scoter, 3,999 ddRAD loci were analyzed for surf scoter, and 3,487 ddRAD loci were analyzed for white‐winged scoter

Within North America, pairwise estimates of genomic structure based on ddRAD loci were low across all species, but comparisons within the black scoter were higher relative to the other scoter species (*Φ*_ST_ < 0.019; Figure 3). Loci with elevated estimates of *Φ*_ST_ (>0.2) were observed for all North American scoters, with the highest proportion observed for comparisons involving black scoters breeding in Alaska and surf scoters breeding in the Central region (Figure 4). Low sample size for the surf scoters samples in the Central region (*N* = 3), however, is likely driving those high proportions. High genomic differentiation was estimated among North American locales for black scoter and common scoter (*Φ*_ST_ = 0.196–0.120) and among white‐winged and velvet (*Φ*_ST_ = 0.155–0.160) scoters (Figure 3).

**Figure A1:**
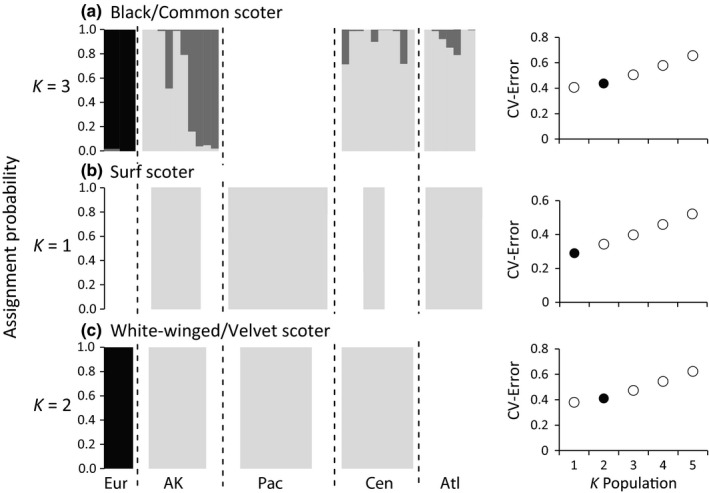
Average assignment probabilities of black/common scoter, surf scoter, and white‐winged/velvet scoter individuals from sampled regions (AK—Alaska; Pac—Pacific; Cen—Central; Atl—Atlantic; Eur—Europe) into one to three clusters inferred from 2,224 ddRAD loci analyzing a single randomly selected biallelic single nucleotide polymorphic site per locus in ADMIXTURE (Alexander & Lange, 2011; Alexander et al., 2009). A plot of cross‐validation (CV) error estimate for each *K* is shown

**Figure A2:**
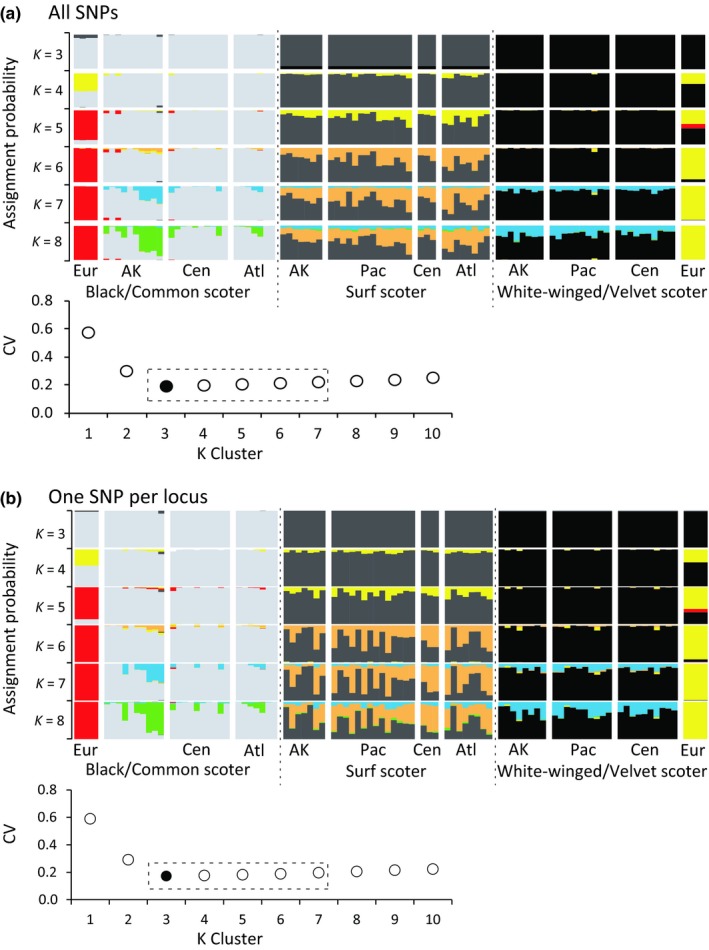
Average assignment probabilities of black/common scoter, surf scoter, and white‐winged/velvet scoter individuals from sampled regions (AK—Alaska; Pac—Pacific; Cen—Central; Atl—Atlantic; Eur—Europe) into three to eight clusters inferred from 2,224 ddRAD loci analyzing (a) all biallelic single nucleotide polymorphic sites (SNP) and (b) a single randomly selected biallelic single nucleotide polymorphic site per locus in ADMIXTURE (Alexander & Lange, 2011; Alexander et al., 2009). Plots of cross‐validation error (CV) estimate for each *K* are shown

All possible *K* values were explored across ADMIXTURE analyses (see Table 2 for total biallelic SNPs per analysis). Analyses based on all biallelic SNPs and a random single SNP per locus yielded similar results (Figure 3; Figures A1, A2). Concordant with *Φ*_ST_ estimates, common scoters and velvet/white‐winged scoters were clearly differentiated from their North American counterparts, black scoters (*K* = 3) and white‐winged scoters (*K* = 2), respectively (Figure 3; Figure A1, A2). Whereas white‐winged scoters showed no structure within North America, black scoter samples separated into two genetic clusters in North America. Specifically, one cluster was predominantly represented by birds sampled in Alaska (Figure 3). This further corresponds with *Φ*_ST_ estimates that show Alaskan birds as more differentiated from the other two locations (*Φ*_ST_ = 0.017–0.019) as compared to black scoters from the Central and Atlantic regions (*Φ*_ST_ = 0.001). Finally, regardless of *K* value, surf scoters showed no structure across North American sampling sites (*Φ*_ST_ < 0.009; Figure 3).

**Figure A3:**
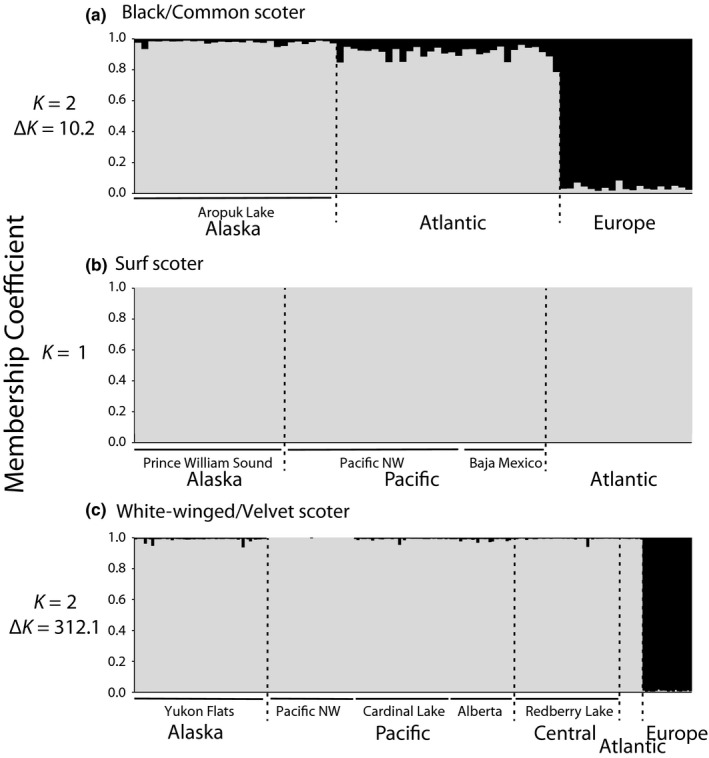
Average membership coefficient and ΔK (*K* = 2) of (a) black/common scoter, (b) surf scoter, and (c) white‐winged/velvet scoter individuals from sampled regions (Alaska, Pacific, Central, and Europe) inferred from 11 microsatellite loci in STRUCTURE (Pritchard et al., 2000). Individuals from the same sample locations are denoted with a black line

**Figure A4:**
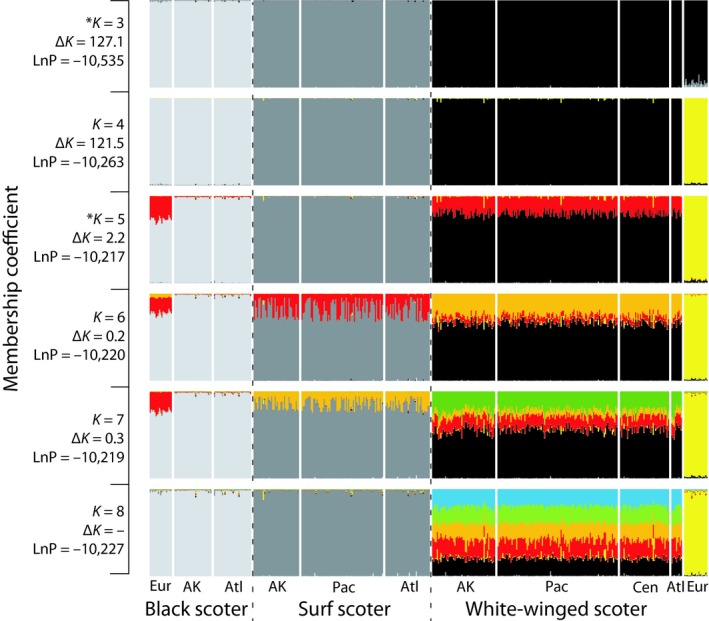
Average membership coefficient, Δ*K*, and average likelihood given the data (LnP) of black/common scoter, surf scoter, and white‐winged/velvet scoter individuals from sampled regions (AK—Alaska; Pac—Pacific; Cen—Central; Atl—Atlantic; Eur—Europe) into three to eight clusters inferred from 11 microsatellite loci in STRUCTURE (Pritchard et al., 2000). The most likely number of clusters based on Δ*K* and LnP are denoted with an asterisk

All microsatellite loci and populations were in HWE and linkage equilibrium. No significant pairwise comparisons were observed within North America based on the microsatellite loci (*F*_ST_ < 0.004; Figure 3) nor were structure detected with the Bayesian clustering analyses (Figure A3). Comparisons among North American locales and European species yielded similar patterns as the ddRAD dataset; high levels of genetic structure were uncovered for both black and common (*F*_ST_ = 0.086–0.102) and white‐winged and velvet (*F*_ST_ = 0.172–0.193) scoters (Figure 3). In addition, black/common scoters and velvet/white‐winged scoter individuals were assigned nearly exclusively to species‐specific clusters (Figure A3), although common scoter individuals clustered with black scoter individuals when all species were analyzed together (Figure A4).

Calculated nucleotide diversity (Table 3) for black (*π* = 0.0031–0.0038) and white‐winged (*π* = 0.0031–0.0036) scoters was slightly higher than those of surf scoters (*π* = 0.0028–0.0029). Diversity metrics were similar between the North American species and their European counterparts. Diversity metrics estimated from the microsatellite loci were similar across regions and species (Table 3).

**Table 3:** Indices of genetic diversity including the mean number of alleles (A), allelic richness (AR), and observed and expected heterozygosity (*H*_o_/*H*_e_) based on 11 microsatellite loci, as well as nucleotide diversity (*π*) calculated using species‐specific and concatenated ddRAD datasets (see Table 2) for black scoter, common scoter^a^, surf scoter, white‐winged scoter, and velvet scoter individuals from sampled regions

	Microsatellites					ddRAD		
	*A*	AR	*H*_o_ (%)	*H*_e_ (%)	*N*	*π* – single species	*π* – combined	*N*
Black scoter								
Alaska	4.6	4.2	54.7	58.5	29	0.0035	0.0034	10
Pacific	–	–	–	–		–	–	
Central	–	–	–	–		0.0032	0.0032	10
Atlantic	5.6	4.8	55.2	60.0	32	0.0031	0.0030	7
Common scoter								
Europe	4.9	4.9	62.5	62.4	19	0.0038	0.0038	5
Surf scoter								
Alaska	3.9	3.9	48.2	47.7	39	0.0029	0.0028	7
Pacific	4.6	4.2	49.4	49.8	68	0.0029	0.0028	14
Central	–	–	–	–		0.0028	0.0028	3
Atlantic	4.6	4.6	46.3	47.4	38	0.0029	0.0029	9
Europe	–	–	–	–		–	–	
White‐winged scoter								
Alaska	5.3	3.3	48.4	49.9	54	0.0036	0.0035	8
Pacific	4.8	3.3	45.1	48.9	40	0.0036	0.0035	10
Central	5.4	3.4	47.0	49.7	42	0.0035	0.0035	10
Atlantic	3.7	3.6	51.5	53.8	9	–	–	
Velvet scoter								
Europe	3.9	3.1	45.5	47.6	20	0.0031	0.0030	4

a Diversity metrics for white‐winged scoter based on microsatellite data are not presented here for nonbreeding and Alberta locales in the Pacific region.

### 3.3 Among‐species comparisons

The traditional species are highly differentiated from each other (BLSC vs. SUSC *Φ*_ST_ = 0.747; BLSC vs. WWSC *Φ*_ST_ = 0.719; SUSC vs. WWSC *Φ*_ST_ = 0.574) as well as between European species (COSC vs. VESC *Φ*_ST_ = 0.730) based on the ddRAD data. Similar levels of structure were observed at microsatellite loci (BLSC vs. SUSC *F*_ST_ = 0.419, *R*_ST_ = 0.515; BLSC vs. WWSC *F*_ST_ = 0.420, *R*_ST_ = 0.689; SUSC vs. WWSC *F*_ST_ = 0.433, *R*_ST_ = 0.541; all *p* < 0.001) and between European species (COSC vs. VESC *F*_ST_ = 0.429, *R*_ST_ = 0.684; *p* < 0.001). No identifiable hybrids between species were found in either the ddRAD or microsatellite datasets (Figures A2 and A4).

### 3.4 ddRAD dataset comparisons

Estimates of differentiation (*R* ^2 ^= 0.99; *p* < 0.0001) and nucleotide diversity (*R* ^2 ^= 0.98; *p* < 0.0001) were significantly correlated whether analyzing species separately (more loci) or together (fewer loci) (Figure 3; Table 2; Figure A1). Moreover, when analyzing all samples together, ADMIXTURE assignments at a *K* of eight (Figure A2) were concordant with those estimated in single species analyses (Figure 3; Figure A1). The latter results suggest that ADMIXTURE analyses are robust to differences in datasets.

## 4 DISCUSSION

### 4.1 Comparisons within North America

The three North American scoter species exhibit weak or no discernible (ddRAD *Φ*_ST_ < 0.019; microsatellite *F*_ST_ < 0.004) genetic structure across their ranges, suggestive of high gene flow and connectivity among regions. The pattern of high connectivity among regions within North America is similar to patterns found in other sea duck species, such that spatial patterns in genetic variation are often not detected or have a weak signal at autosomal loci (Talbot, Sonsthagen, Pearce, & Scribner, 2015). The lack of spatial genetic structure is most often attributed to male‐biased dispersal playing a role in homogenizing allelic frequencies across the landscape, as pair formation occurs on winter areas where nonbreeding aggregations are formed by individuals from multiple nesting areas (e.g., common merganser, *Mergus merganser*, Pearce, Zwiefelhofer, & Maryanski, 2009, Peters, Bolender, & Pearce, 2012; hooded merganser, *Lophodytes cucullatus*, Pearce, Blums, & Lindberg, 2008; king eider, *Somateria spectabilis*, Pearce et al., 2004; long‐tailed duck, *Clangula hyemalis*, Wilson et al., 2016; spectacled eider, *S. fischeri*, Scribner et al., 2001; Steller's eider, *Polysticta stelleri*, Pearce, Talbot, Petersen, & Rearick, 2005; but see common eider, *S. mollissima*, Sonsthagen et al., 2011). However, male‐mediated dispersal is likely not the sole mechanism promoting genomic connectivity across North America.

Migratory tendencies of female surf scoters and white‐winged scoters may explain, at least in part, the lack of genomic partitioning within North America. White‐winged scoters that nest at Redberry Lake, Saskatchewan, and surf scoters that nest in north central Canada (near Great Slave Lake) are comprised of birds that winter on the Atlantic and Pacific coasts (De La Cruz et al., 2009; Sea Duck Joint Venture, 2015; Swoboda, 2007; Takekawa et al., 2011). Among white‐winged scoters nesting at Redberry Lake, there is strong evidence that at least one female white‐winged scoter (and weaker evidence for five others, *N* = 62) switched between Atlantic and Pacific wintering areas among years (Swoboda, 2007). A similar pattern is observed among king eiders that nest at Karrak Adventure lakes, Nunavut, Canada; the population is comprised of individuals that winter on the Atlantic and Pacific coasts, females switched (*N* = 6/20) winter areas between years (Mehl, Alisauskas, Hobson, & Kellett, 2004), and genetic structure was not detected among eastern and western populations (Pearce et al., 2004). Moreover, factors influencing an individual's migratory tendencies vary by species (i.e., genetic disposition, environmental factors, individual condition, Pulido, 2007; cultural influence, Palacin, Alonso, Alonso, Magana, & Martin, 2011), and a variety of underlying factors likely play a role in the development and maintenance of migratory strategies among individuals. Within populations comprised of individuals with differing migratory affinities, the influence of nongenetic variance components determining migratory behavior (i.e., east vs. west migration) in young birds (i.e., development of eastern migration when parents migrate west) would further homogenize allelic frequencies among regions. As pair formation occurs either on the wintering areas (i.e., surf scoter) or during spring migration (i.e., white‐winged scoter), any reduction in fidelity to winter areas or variation in migratory behavior by females and their young provides an avenue for intracontinental dispersal by males, thereby homogenizing allelic frequencies within the nuclear genome.

The lack of phylogeographic structure within white‐winged scoters is particularly interesting because this species pairs during spring migration or summer, and therefore, we would expect some evidence of structure among regions as evident in other waterfowl species that share this characteristic (e.g., greater white‐fronted goose, Wilson et al., 2018). Summer pairing would also be conducive of multiyear pairing and has been proposed (although not confirmed) for white‐winged and surf scoters (Anderson et al., 2015; Brown & Fredrickson, 1997). Pair formation during spring migration or summer would restrict the availability of mates. However, recent studies using satellite telemetry found that two females used different spring migration routes between years (Meattey et al., 2018) and that male white‐winged scoters migrated to different breeding areas between years (Sea Duck Joint Venture, 2015). If these behaviors are common in white‐winged scoters, they may enable the formation of pair bonds between individuals from different breeding areas, serving to homogenize allele frequencies across nesting locales.

Population models generated to assess population declines in white‐winged scoters nesting on Redberry Lake led the researchers to hypothesize that the population was rescued by female immigration (Alisauskas, Traylor, Swoboda, & Kehoe, 2004). A rescue (source–sink) dynamic via female dispersal mitigating population decline was also postulated to occur among nesting areas of spectacled eiders within the Yukon‐Kuskokwim Delta, Alaska (Flint, Grand, Petersen, & Rockwell, 2016), and Chaun Delta, Chukotka (Solovyeva, Vartanyan, Frederiksen, & Fox, 2018). These studies indicate that females disperse among nesting areas (at least among areas that are experiencing decline), despite the presumption of high female philopatry within sea duck species (Eadie & Savard, 2015). The lack of genomic structure across North America indicates that intracontinental gene flow is occurring for both surf scoter and white‐winged scoter. We assayed only putative autosomal loci and therefore cannot differentiate between male‐ or female‐biased dispersal; data from maternally inherited markers are needed to confirm this hypothesis of female dispersal. Given that sea duck species share similar life‐history characteristics, it seems likely that male‐biased gene flow is also influencing the levels of genomic structure observed among regions within scoters.

Phylogeographic structure observed within black scoters at ddRAD loci is concordant with the disjunct breeding distribution. The barrier, whether behavioral or physical, between Alaska and Central/Atlantic regions for black scoters appears to limit dispersal among areas as evidenced by the 17‐fold higher *Φ*_ST_ between segregated regions. Arctic and sub‐Arctic taxa often exhibit a phylogeographic break located near the Mackenzie River, western Canada, which represents the eastern boundary of Beringia (Hewitt, 2004b); this signature is present, although subtle (ddRAD *Φ*_ST_ = 0.017–0.019) within black scoter and not detected at microsatellite loci (Figure 3a). Among sea duck species for which autosomal data are available (Pearce et al., 2004, 2005, 2008; 2009; Peters et al., 2012; Scribner et al., 2001; Sonsthagen et al., 2011; Wilson et al., 2016), only the common eider exhibits a similar break in genetic structure among birds that winter in the Pacific versus Atlantic oceans (microsatellite *F*_ST_ = 0.094, nuclear introns *Φ*_ST_ = 0.098–0.120; Sonsthagen et al., 2011). Maintenance of genetic structure among regions in common eider was attributed to high female philopatry coupled with winter site fidelity, as partitions in the nuclear genome correspond to subspecific designations for the species (Sonsthagen et al., 2011). While we only assayed autosomal loci and therefore cannot specifically hypothesize about female scoter dispersal tendencies, the spatial pattern of genomic diversity in black scoter suggests that the species may exhibit higher levels of winter site fidelity, which would decrease opportunities for pair‐bonding with individuals from other nesting locations (i.e., lower incidence of intracontinental dispersal) than the other scoter species.

Finally, Holocene response following Pleistocene glacial cycling may also play a role in the shallow differentiation of populations of scoters. Most of northern North America was covered by the Cordilleran and Laurentide ice sheets through the last glacial maximum, and only recently colonized as species’ distributions expanded into habitat made newly available by glacial retreat (Hewitt, 2004a). As scoters have only likely recently occupied northern North America (<11,000 years), insufficient time may have passed to accumulate regional level differences at nuclear markers (i.e., incomplete lineage sorting). Indeed, only a small percentage of loci (<3.9%) recovered exhibited elevated levels of divergence, which can be attributed to incomplete lineage sorting or dispersal. Both processes (dispersal and recent divergence), therefore, are likely playing a role in patterns of genetic diversity observed within scoters sampled across North America.

### 4.2 Comparisons between continents

North American species are highly differentiated from their European counterparts (*Φ*_ST_ = 0.196–0.200, *F*_ST_ = 0.086–0.102 black/common scoter; *Φ*_ST_ = 0.155–0.160, *F*_ST_ = 0.172–0.193 white‐winged/velvet scoter), with no evidence of intercontinental gene flow based on ADMIXTURE (Figure 3) or STRUCTURE plots (Figure A2). The genomic partition within white‐winged/velvet scoter is particularly interesting as DNA barcoding did not uncover intercontinental structure (Johnsen et al., 2010). The pattern of high genomic partitioning at nuclear loci between North American and European forms was uncovered in other sea duck species (common eider microsatellite *F*_ST_ = 0.000–0.166, nuclear introns *Φ*_ST_ = 0.000–0.208; Sonsthagen et al., 2011; common merganser nuclear introns *Φ*_ST_ = 0.254–0.274; Peters et al., 2012); comparisons among locales with overlapping winter ranges were low and attributed to male‐biased dispersal (Sonsthagen et al., 2011; see also Scribner et al., 2001). The general pattern of high genetic structure at nuclear loci between North American and European forms in sea ducks is unique in waterfowl. Among other waterfowl species, intercontinental estimates of genetic structure (*F*_ST_) were below 0.090 with incomplete lineage sorting posited for lack of partitioning observed (exception *Anas acuta*: inference was high gene flow, Flint et al., 2009; see table S1 in Peters et al., 2012 for *A. acuta*, *Mareca strepera*, formally *Anas*, and *Spatula cyanoptera*, formally *Anas*; *A. platyrhynchos*, Kraus et al., 2013; *Anser albifrons*, Wilson et al., 2018). As most sea duck species have a Holarctic distribution (or closely related conspecifics which together form a Holarctic distribution), isolation in Arctic refugia may have promoted the formation of North American and European varieties. Indeed, inferences of historical population demography identified four areas where common eiders were likely restricted during the Last Glacial Maximum (Belcher Islands and Newfoundland Bank, Canada; northern Alaska, USA; and Svalbard, Norway), which coincide with previously identified refugia: Newfoundland Bank, Beringia, and Spitsbergen Bank (Sonsthagen et al., 2011). Genomic partitions are likely maintained by nonoverlapping (or nearly so) winter distributions (Collinson, Parkin, Knox, Sangster, & Helbig, 2006), which could limit opportunities to form pair bonds among North American and European varieties and ultimately restrict intercontinental dispersal. Examination of range‐wide genomic structure among the other sea duck species (king eider, common goldeneye, long‐tailed duck, and red‐breasted merganser) is needed to confirm whether this pattern of high genomic structure between North American and Eurasian forms is a general characteristic of sea ducks, or unique to the scoters, common eider, and common merganser.

### 4.3 Hybridization

Waterfowl are known for their propensity to hybridize with prezygotic mechanisms (e.g., courtship displays and vocalizations) maintaining species boundaries. Due to the high rate of genomic connectivity found within sympatric dabbling ducks (genus *Anas*), Kraus et al. (2012) coined the term “suprapopulation” where a group of species form a superspecies complex where natural hybridization occurs but without eroding species barriers. Scoter species are known to hybridize with each other; however, we did not detect any evidence of hybridization or introgression within or among traditional species (Figure 3), despite the traditional species pairs (white‐winged/velvet scoter and black/common scoter) demonstrating similar courtship displays (Collinson et al., 2006). Although our sample sizes for ddRAD analyses are small for the European forms, limiting our inferences, we failed to detect introgression among the three North American species based on our larger microsatellite genotype dataset (*N* = 457; see also Talbot et al., 2015). Courtship and copulation displays among black/common, velvet/white‐winged, and surf scoters are diagnostic, perhaps providing a behavioral barrier to hybridization among the three traditionally accepted species (Collinson et al., 2006). Thus, although hybridization has been detected (white‐winged scoter x surf scoter), our data support the supposition that these events are rare (Brown & Fredrickson, 1997) and do not appear to result in excessive introgression.

### 4.4 Conservation implications

Scoters are migratory species that nest in remote regions of the Arctic, have experienced population declines, and are game species. Furthermore, certain characteristics of scoter breeding biology (low reproductive output, delayed breeding, etc.) may limit their capacity to recover from population declines and events (either stochastic or deterministic) that reduce adult survival (Koneff et al., 2017). Understanding linkages among key stages in the annual cycle are important to inform management strategies and conserve species, as events (i.e., disease, habitat quality, nutrition, and weather) during the nonbreeding season affect an individual's body condition, survival, and fecundity (Sedinger & Alisauskas, 2014). However, studies that investigate the level of population connectivity via dispersal in scoters (and waterfowl in general) must also contend with the lack of complete understanding about the relationship between migratory and dispersal behavior. Added to this layer of complexity is the fact that most waterfowl undertake a postbreeding remigial molt, where males, some nonbreeders, and failed female breeders migrate elsewhere to molt. Despite the lack of (or weak) genomic structure observed within North America that suggests that scoter populations are connected through a dispersal network that facilitates and/or maintains panmixia, high‐resolution loci (*Φ*_ST_ > 0.20) among regions were uncovered (Figure 4). Analysis of these high‐resolution loci assayed from nesting, molting, and wintering areas may provide an opportunity for researchers to further elucidate linkages among areas within the annual cycle (e.g., Ruegg et al., 2014) and provide insights on the composition of nesting areas among hunter‐harvested individuals, enabling managers to develop harvest prescriptions that serve to reduce pressure on populations experiencing declines.

## CONFLICT OF INTEREST

None declared.

## AUTHORS’ CONTRIBUTION

All authors conceived of the project, collected the data, performed the analyses, and contributed to the writing of the manuscript.

## Data Availability

The data used in the present study are accessioned in NCBI Sequence Read Archive (BioProject PRJNA541567, accessions SAMN11587829–115187923) and in Sonsthagen et al. (2019).
